# Real‐World Data of Retroperitoneal Tumor Surgeries Performed by Gastroenterological Surgeons in Japan: Analysis Based on the National Clinical Database

**DOI:** 10.1002/ags3.70204

**Published:** 2026-02-24

**Authors:** Keisuke Kurimoto, Hiroyuki Yamamoto, Masaki Sunagawa, Yoshiki Kajiwara, Hiroshi Hasegawa, Hideki Ueno, Ken Shirabe, Mitsuro Kanda, Tomoki Ebata, Yukihiro Yokoyama

**Affiliations:** ^1^ Department of Surgery Nagoya University Hospital Nagoya Aichi Japan; ^2^ Department of Healthcare Quality Assessment, Graduate School of Medicine The University of Tokyo Tokyo Japan; ^3^ Project Management Subcommittee The Japanese Society of Gastroenterological Surgery Tokyo Japan; ^4^ Department of Surgery National Defense Medical College Saitama Japan; ^5^ Division of Gastrointestinal Surgery, Department of Surgery Kobe University Graduate School of Medicine Kobe Hyogo Japan; ^6^ Database Committee The Japanese Society of Gastroenterological Surgery Tokyo Japan; ^7^ The Japanese Society of Gastroenterological Surgery Tokyo Japan; ^8^ Division of Hepatobiliary and Pancreatic Surgery, Department of General Surgical Science, Graduate School of Medicine Gunma University Maebashi Gunma Japan

**Keywords:** national clinical database (NCD), rare cancers, real‐world data, retroperitoneal tumor, surgical outcomes

## Abstract

**Background:**

Retroperitoneal tumors (RPTs) are rare and anatomically complex neoplasms, for which surgery remains the mainstay of treatment. However, real‐world data on their surgical management in Japan have been limited.

**Objective:**

To describe the clinical characteristics and short‐term outcomes of patients undergoing resection for RPTs in Japan, based on data from gastroenterological surgical practice.

**Methods:**

This study analyzed data from the Japanese National Clinical Database (NCD) for gastroenterological surgery. A total of 4948 patients with RPT who underwent surgery between 2019 and 2021 were included.

**Results:**

There were 2360 men (47.7%) and 2588 women (52.3%), with a median age of 66 years. RPTs were histologically classified as malignant in 75.3% and benign in 24.7% of cases. The median operative time was 205 min, and the median blood loss was 150 mL. Postoperative complications occurred in 23.9% of patients, with 7.5% experiencing severe complications (Clavien–Dindo grade III or higher). The 30 day postoperative mortality rate was 0.5%, and the perioperative mortality rate was 1.0%.

**Conclusion:**

This analysis demonstrates that a substantial number (approximately 1650 per year) of RPTs surgeries are performed annually by gastrointestinal surgeons in Japan, and that the short‐term surgical outcomes are acceptable. These data provide an important reference to exhibit the current surgical practice in Japan and to develop future strategies for RPTs.

## Abstract 2

We analyzed real‐world data from the National Clinical Database to describe surgical management of retroperitoneal tumors performed by gastroenterological surgeons in Japan. Among 4948 surgical cases between 2019 and 2021, malignant tumors predominated, and postoperative complications occurred in approximately one‐quarter of patients, with low perioperative mortality. These findings demonstrate that, despite the complexity of retroperitoneal tumor surgery, short‐term outcomes in contemporary Japanese practice are acceptable.

## 1 Introduction

Retroperitoneal tumors (RPTs) are relatively rare disease entity that develop in the retroperitoneal space, involving tissues such as adipose tissue, muscle, lymphatic structure, nerves, and major blood vessels. Their incidence is extremely low—reportedly 2.7 cases per 1 000 000 persons according to data from the SEER (Surveillance Epidemiology and End Results) program in the United States [1]. In Japan, malignant RPTs are classified as rare cancers, with an estimated annual incidence of approximately 6.3 per 1 000 000 population [2] (https://ganjoho.jp/public/qa_links/report/statistics/pdf/cancer_statistics_2025_data_J.pdf?utm_source=chatgpt.com). Despite their rarity, opportunities to encounter patients with RPTs are not uncommon; indeed, many general surgeons have some experience in their management.

RPTs represent a highly heterogeneous group of tumors that vary widely in origin, anatomical extent, histopathology, and malignant potential [3, 4]. Moreover, because these tumors can involve or extend across multiple anatomical regions, their surgical management often requires multidisciplinary collaboration among general surgeons, urologists, gynecologists, orthopedic surgeons, and vascular surgeons.

Most of the existing literature on RPTs consists of case reports or single‐institution series with limited sample sizes [1, 5, 6, 7, 8], and epidemiological data on their actual clinical management remain scarce [9, 10]. Particularly in Japan, the lack of comprehensive real‐world data has precluded a nationwide assessment of surgical cases of RPTs, thereby hindering the evaluation of treatment outcomes and perioperative risks at a population level. In Japan, a nationwide cancer registry system exists based on the Cancer Registry Act and other related frameworks, enabling the collection of incidence and survival data for major tumor types. However, as for RPTs, the rarity and extensive heterogeneity have limited our ability to evaluate not only treatment outcomes but also the accumulation of epidemiological data. The National Clinical Database (NCD) is a nationwide surgical registry maintained by the Japan Surgical Society and affiliated organizations, encompassing data from approximately 6000 participating institutions. In addition to its high coverage rate [11, 12], the NCD includes registration schemes of rare diseases including RPTs. Accordingly, the NCD, which collects approximately 1.5 million surgical cases annually, provides a more suitable data source than cancer registries for characterizing the real‐world surgical practice for RPTs in Japan.

In this study, we aimed to provide a nationwide descriptive overview of the real‐world clinical characteristics and short‐term surgical outcomes of RPTs in Japan, using data from the NCD for gastroenterological surgery. This large‐scale, retrospective analysis is expected to offer valuable insights into the status and challenges of managing this rare and heterogeneous disease.

## 2 Patients and Methods

We extracted data on patients who underwent surgery for RPTs between January 1, 2019, and December 31, 2021, from the gastroenterological surgery subset of the NCD. Surgical procedures were identified using the Japanese surgical coding system, including the following codes: OQ0094 (omental, mesenteric, and retroperitoneal tumor removal without intestinal resection), OQ0095 (omental, mesenteric, and retroperitoneal tumor removal with intestinal resection), OZ0008 (retroperitoneal malignant tumor simple removal), OZ0009 (retroperitoneal malignant tumor radical resection), SZ0010 (laparoscopic retroperitoneal tumor resection), and NQ0755 (multiple organ resection with peritoneal resection) (Table S1). The NCD allows multiple surgical codes to be assigned to a single patient.

From the database, we extracted the following variables: age at the time of surgery, sex, preoperative American Society of Anesthesiologists physical status (ASA‐PS) classification, preoperative treatment status (chemotherapy, radiotherapy, immunotherapy), operative time, blood loss, need for blood transfusion, postoperative complications classified according to the Clavien–Dindo system, condition at 30 days postoperatively, readmission within 30 days, reoperation within 30 days, and discharge disposition. Perioperative mortality was defined as death recorded either as “death” in the discharge disposition or as “death” in the 30 day postoperative status in the database.

This study was approved by the Japanese Society of Gastroenterological Surgery and the institutional review board of Nagoya University (approval no. 2022–023626582). This study was conducted in accordance with the outlined in the provisions of the Declaration of Helsinki.

## 3 Results

### 3.1 Number of Surgeries

**FIGURE 1:**
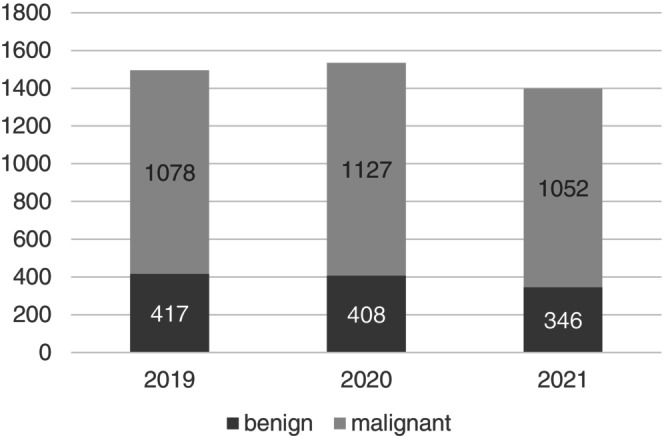
Number of surgeries. The figure shows the total number of surgeries performed each year between 2019 and 2021 for retroperitoneal tumors.

A total of 4948 retroperitoneal tumor surgeries were registered in the NCD for gastroenterological surgery between 2019 and 2021. Among these, 3726 cases (75.3%) were histologically classified as malignant tumors and 1222 cases (24.7%) as benign tumors. The annual number of cases remained consistent throughout the study period (Figure S1) (Figure 1) shows the annual distribution of cases and the proportion of malignant tumors.

The most frequently recorded surgical procedure was OZ0008, followed by OQ0094 and OZ0009. Laparoscopic procedures (SZ0010) were predominantly performed for benign tumors. The distribution of procedures by tumor classification is shown in (Table S1).

### 3.2 Patient Characteristics

Regarding patient demographics (Table 1), the sex distribution was approximately equal between the benign group and malignant group.

**TABLE 1:** Patient characteristics.

	Total (*n* = 4948)	Benign (*n* = 1222)	Malignant (*n* = 3726)
Sex, male (%)	2360 (47.7)	526 (43.0)	1834 (49.2)
Age, years [IQR]^a^	66 [53, 73]	57 [45, 70]	67 [56, 74]
ASA‐PS^b^ ≤ 2 (%)	4365 (88.2)	1108 (90.7)	3257 (87.4)
Preoperative chemotherapy (%)	517 (10.4)	7 (0.6)	510 (13.7)
Preoperative radiotherapy (%)	46 (0.9)	1 (0.1)	45 (1.2)
Preoperative immunotherapy (%)	10 (0.2)	6 (0.5)	4 (0.1)

*Note:* This table summarizes demographic and preoperative clinical characteristics of patients undergoing surgery for retroperitoneal tumors between 2019 and 2021, stratified into benign and malignant groups.

^a^ IQR: Interquartile Range.

^b^ American Society of Anesthesiologists physical status.

**FIGURE 2:**
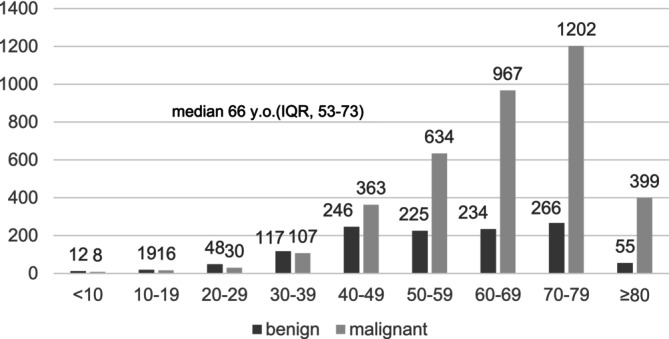
Number of surgeries by age. Age distribution of patients undergoing surgery for retroperitoneal tumors, grouped by 10 year intervals.

The overall median age was 66 years (interquartile range [IQR]: 53–73 years). Patients with malignant tumors had a median age of 67 years (IQR: 56–74), whereas those with benign tumors were younger, with a median age of 57 years (IQR: 45–70). Among malignant cases, the number of surgeries increased with age, whereas benign tumor surgeries were relatively evenly distributed after age 40. Notably, 16% of patients with benign tumors were under 40 years old, compared with only 4.3% in the malignant group (Figure 2).

The number of surgeries performed in patients aged 80 years or older was low, regardless of tumor malignancy.

The majority of patients were in good general condition, with 88.2% having an ASA‐PS of ≤ 2. Preoperative chemotherapy was administered to 13.7% of patients with malignant tumors (*n* = 510), whereas preoperative radiotherapy and immunotherapy were rarely used.

### 3.3 Surgical Variables

The median operative time was 198 min (interquartile range [IQR]: 125–301) (Table 2). In cases of benign tumors, the median operative time was 154 min (IQR: 112–229), whereas in malignant tumors it was 218 min (IQR: 135–321).

**TABLE 2:** Intraoperative characteristics.

	Total (*n* = 4948)	Benign (*n* = 1222)	Malignant (*n* = 3726)
Operation time, min [IQR]^a^	198 [125, 301]	154 [112, 229]	218 [135, 321]
Blood loss, ml [IQR]^a^	150 [20, 615]	50 [5, 218]	215 [33, 790]
Transfusion (%)	859 (17.4)	78 (6.4)	781 (21.0)

*Note:* This table presents operative findings for patients with retroperitoneal tumors, compared to benign and malignant tumors.

^a^ IQR: Interquartile Range.

The overall median estimated blood loss was 150 mL (IQR: 20–615). The median blood loss was 50 mL for benign tumors, whereas it was 215 mL for malignant tumors. Operative time and blood loss by each surgical procedure are shown in Table S2. The three procedures involving bowel resection, extensive excision, or combined resection of multiple organs (OQ0095, OZ0009, and NQ0755) were associated with long operative times and large volumes of blood loss.

Malignant tumors were also associated with a higher rate of blood transfusion than benign tumors (21% vs. 6.4%).

### 3.4 Postoperative Complications and Outcomes

Overall, postoperative complications occurred in 1183 patients (23.9%) (Table 3). Among them, 371 patients (7.5%) experienced complications classified as Clavien–Dindo grade III or higher. The complication rate was 16.4% in patients with benign tumors and 26.4% in those with malignant tumors. Severe complications, defined as Clavien–Dindo grade III or higher, occurred in 3.8% of benign cases and 8.7% of malignant cases. Postoperative outcomes according to surgical procedure are shown in Figure S2. In the majority of cases, no postoperative complications were observed across all surgical procedures. Postoperative outcomes are shown in (Table 3). There were 23 deaths within 30 days after surgery, accounting for 0.5% of all cases. A total of 167 patients (3.4%) were readmitted within 30 days, and 161 patients (3.3%) underwent reoperation during the same period. When including patients whose discharge disposition was recorded as “death” in addition to those who died within 30 days, the total number of perioperative deaths was 49, accounting for 1.0% of all cases. The majority of perioperative deaths occurred in patients with malignant tumors.

**TABLE 3:** Postoperative outcomes.

	Total (*n* = 4948)	Benign (*n* = 1222)	Malignant (*n* = 3726)
Postoperative complications (the Clavien‐Dindo classification)			
Grade 0	3765 (76.1)	1021 (83.6)	2744 (73.6)
Grade I	366 (7.4)	74 (6.1)	292 (7.8)
Grade II	466 (9.0)	80 (6.5)	366 (9.8)
≥ Grade III	371 (7.5)	47 (3.8)	324 (8.7)
Mortality within 30 days	23 (0.5)	1 (0.1)	22 (0.6)
Readmission within 30 days	167 (3.4)	23 (1.9)	144 (3.9)
Reoperation within 30 days	161 (3.3)	23 (1.9)	138 (3.7)
Perioperative mortality	49 (1.0)	3 (0.2)	46 (1.2)

*Note:* This table shows short‐term postoperative outcomes, including postoperative complications classified according to the Clavien–Dindo classification, 30 day mortality, readmission, reoperation, and perioperative mortality, separately for benign and malignant retroperitoneal tumors.

## 4 Discussion

Our analysis highlights that although malignant RPTs are classified as rare cancers, surgeons in Japan occasionally encounter them in clinical practice, and both morbidity and mortality were within acceptable limits. This study is the first nationwide analysis to describe real‐world surgical trends and outcomes for RPTs in Japan using the NCD data for gastroenterological surgery.

Despite their low prevalence, approximately 1400 to 1500 surgeries for RPTs are performed annually by general surgeons in Japan. According to 2023 data from the NCD, this figure is comparable to the number of pancreaticoduodenectomies with arterial or portal vein reconstruction (NCD code: OQ0272, *n* = 1443). This finding suggests that RPT surgery is not uncommon in clinical practice, despite the rarity of the disease itself.

In this study, approximately 75% of RPT cases were malignant. Because complete surgical resection is the only curative treatment for malignant RPTs, it is reasonable to assume that, except for patients in poor general condition and contraindications to surgery, most patients with malignant RPTs underwent resection [13, 14]. Therefore, the NCD cohort may reasonably reflect the real‐world situation of malignant RPTs in Japan. In contrast, benign lesions are frequently managed without intervention unless symptomatic or radiologically indistinguishable from malignancy. As the NCD includes only surgical cases, it does not capture non‐operative cases, and thus, cannot provide true incidence rates of benign RPTs. Nevertheless, these data provide a comprehensive snapshot of the current surgical landscape for RPTs in Japan.

Demographically, malignant tumors were more prevalent among older adults, whereas benign tumors were more common in younger patients, with 16% occurring in individuals under the age of 40. Conversely, the number of surgeries in patients aged 80 years and older declined, suggesting a tendency to avoid surgical intervention in this age group, likely due to comorbidities and increased surgical risk. The fact that most patients had an ASA‐PS of 2 or less indicates that most had relatively good preoperative health. However, previous reports have suggested that although the incidence of postoperative complications increases in elderly patients undergoing RPT surgery, neither perioperative mortality nor overall survival is significantly affected [15, 16]. In the present analysis, age‐stratified evaluation of postoperative complications did not demonstrate a marked increase in overall or severe complications among patients aged 60 years and older, including those aged 80 years and above (Table S3). Taken together, these findings suggest that avoidance of surgery in elderly patients solely due to chronological age may need to be reconsidered.

Operative characteristics showed that malignant tumors required longer operative times, greater blood loss, and higher transfusion rates. This is likely explained by the frequent need for combined organ resections and the invasive nature of malignant tumors. Laparoscopic surgery was primarily used for benign tumors, reflecting a lower level of surgical invasiveness in these cases. These differences in surgical approach and complexity align with tumor biology and clinical behavior.

Postoperative complications occurred in 23.9% of all patients, with severe complications occurring in 7.5%. The rate was higher among patients with malignant tumors (8.7%), but even among benign cases, 3.8% experienced significant complications, underscoring the need for careful perioperative management regardless of tumor type. Most reoperations and deaths occurred in the malignant group, highlighting the higher risk associated with complex and extensive procedures. However, the overall 30 day mortality rate was only 0.5%, reflecting acceptable safety in the surgical management of these challenging tumors. Previous population‐based and multicenter studies of retroperitoneal tumor surgery have reported major postoperative complication rates of approximately 15%–20% and perioperative mortality rates ranging from 1%–5%. In this context, the perioperative outcomes observed in the present study fall well within the range of previously published data [17, 18, 19, 20]. Future research should focus on integrating histological and long‐term outcomes, as well as evaluating the centralization of care in Japan and multidisciplinary strategies to improve patient outcomes [6, 21, 22, 23, 24, 25, 26, 27, 28].

## 5 Limitation

This study has several limitations inherent to the use of registry data. First, the NCD primarily includes surgeries performed by gastrointestinal surgeons, and procedures conducted by other specialties such as urology, gynecology, or orthopedic surgery may not be captured. Therefore, the full spectrum of RPT surgeries in Japan may be underrepresented. Second, the database includes only patients who underwent surgery, excluding those managed non‐operatively or deemed unresectable. Third, detailed tumor‐specific information is not available in the NCD. Variables such as tumor histology, anatomical location, invasion of adjacent structures, recurrence status, and long‐term outcomes are not recorded. This limitation hinders the ability to perform oncologic analyses or evaluate prognostic factors.

To address these limitations and gain a more accurate understanding of the current status of RPTs and their surgical management in Japan, future linkage of the National Clinical Database with population‐based cancer registries, or extension of the NCD to include tumor histology and long‐term outcomes, could help overcome these limitations and enable more comprehensive oncologic analyses. Such a system would contribute to improving treatment outcomes for RPTs, which are classified as rare cancers.

## 6 Conclusion

This nationwide analysis of RPT surgeries using the NCD provides important insights into the epidemiology, surgical practices, and short‐term outcomes of this rare and heterogeneous group of tumors. Although RPTs are uncommon, a substantial number of surgical cases are performed annually in Japan, with malignant tumors accounting for the majority.

RPTs present anatomical challenges and are often associated with high surgical invasiveness; however, the low perioperative mortality observed in carefully selected patients supports the safety and feasibility of surgery in this population.

Future studies incorporating tumor biology and long‐term outcomes will further strengthen the evidence base for treatment strategies in RPTs.

## Author Contributions

**Keisuke Kurimoto:** project administration, writing – original draft, conceptualization, writing – review and editing, methodology. **Hiroyuki Yamamoto:** writing – original draft, conceptualization, writing – review and editing, data curation, methodology, project administration. **Masaki Sunagawa:** writing – review and editing. **Yoshiki Kajiwara:** conceptualization. **Hiroshi Hasegawa:** conceptualization. **Hideki Ueno:** writing – review and editing. **Ken Shirabe:** writing – review and editing. **Mitsuro Kanda:** writing – review and editing. **Tomoki Ebata:** writing – review and editing. **Yukihiro Yokoyama:** writing – original draft, conceptualization, methodology, project administration, writing – review and editing.

## Funding

The authors have nothing to report.

## Disclosure

Hiroyuki Yamamoto is affiliated with the Department of Healthcare Quality Assessment at the University of Tokyo. This department is a social collaboration department supported by grants from the National Clinical Database, Intuitive Surgical Sarl, Johnson & Johnson K.K., and Nipro Co. (As noted in the manuscript).

Dr. Hideki Ueno, Dr. Ken Shirabe and Dr. Mitsuro Kanda are the Editorial Board members of ASES Journal and the co‐authors of this article. To minimize bias, they were excluded from all editorial decision‐making related to the acceptance of this article for publication.

## Ethics Statement

This study was approved by the ethical review boards of Nagoya University (approval no, 2022–023626582) (As noted in the manuscript).

## Consent

The requirement for written informed consent was waived because of the retrospective study design and the use of anonymized clinical data (As noted in the manuscript). Information regarding the opt‐out process is available on the website of the Japanese Society of Gastroenterological Surgery (https://www.jsgs.or.jp/career/ncd/ncd‐study/).

## Conflicts of Interest

The authors declare no conflicts of interest.

## Supporting information


**Figure S1:** Number of surgeries by age group per year.
Temporal trends in the number of surgeries stratified by age group over the study period (2019–2021).
**Figure S2:** Postoperative complication rates by surgical procedure.
Comparison of postoperative complication rates across six major surgical procedures.

## References

[1] G. A. Porter , N. N. Baxter , and P. W. Pisters , “Retroperitoneal Sarcoma: A Population‐Based Analysis of Epidemiology, Surgery, and Radiotherapy,” Cancer 106, no. 7 (2006): 1610–1616. 16518798 10.1002/cncr.21761

[2] Foundation for Promotion of Cancer Research [Internet] , Cancer Statistics in Japan‐2025 [Internet] (Foundation for Promotion of Cancer Research, 2025), https://ganjoho.jp/public/qa_links/report/statistics/pdf/cancer_statistics_2025_data_J.pdf.

[3] D. C. Strauss , A. J. Hayes , and J. M. Thomas , “Retroperitoneal Tumours: Review of Management,” Annals of the Royal College of Surgeons of England 93, no. 4 (2011): 275–280. 21944791 10.1308/003588411X571944 PMC3363075

[4] Y. Yokoyama and Y. Kodera , “Diagnosis and Treatment of Retroperitoneal Tumors,” J Jpn Surg Assoc 81, no. 4 (2020): 623–635.

[5] A. Buja , M. Rugge , M. Barillaro , et al., “Epidemiology, Pathological Characteristics and Survival of Retroperitoneal Soft‐Tissue Sarcomas Compared With Non‐Retroperitoneal Soft Tissue Sarcomas,” Oncology Letters 26, no. 1 (2023): 301. 37323817 10.3892/ol.2023.13887 PMC10265397

[6] O. Peacock , S. Patel , J. A. Simpson , C. J. Walter , and D. J. Humes , “A Systematic Review of Population‐Based Studies Examining Outcomes in Primary Retroperitoneal Sarcoma Surgery,” Surgical Oncology 29 (2019): 53–63. 31196494 10.1016/j.suronc.2019.03.002

[7] C. A. Stiller , A. Trama , D. Serraino , et al., “Descriptive Epidemiology of Sarcomas in Europe: Report From the RARECARE Project,” European Journal of Cancer 49, no. 3 (2013): 684–695. 23079473 10.1016/j.ejca.2012.09.011

[8] J. C. F. Willburger , M. von Strauss , C. J. Peterson , T. R. Glass , and C. Kettelhack , “Incidence, Treatment and Outcome of Patients With Retroperitoneal Soft‐Tissue Sarcoma in Switzerland 2005‐2015: A Population‐Based Analysis,” World Journal of Surgery 46, no. 2 (2022): 461–468. 34755196 10.1007/s00268-021-06374-z PMC8724195

[9] M. A. Clark , C. Fisher , I. Judson , and J. M. Thomas , “Soft‐Tissue Sarcomas in Adults,” New England Journal of Medicine 353, no. 7 (2005): 701–711. 16107623 10.1056/NEJMra041866

[10] A. M. Villano , A. Zeymo , K. S. Chan , K. R. Unger , N. Shara , and W. B. Al‐Refaie , “Variations in Retroperitoneal Soft Tissue Sarcoma Outcomes by Hospital Type: A National Cancer Database Analysis,” JCO Oncol Pract 16, no. 9 (2020): e991–e1003. 32267809 10.1200/JOP.19.00460

[11] Y. Kajiwara , A. Takahashi , H. Ueno , et al., “Annual Report on National Clinical Database 2020 for Gastroenterological Surgery in Japan,” Ann Gastroenterol Surg 7, no. 3 (2023): 367–406. 37152776 10.1002/ags3.12662 PMC10154850

[12] Y. Seto , Y. Kakeji , H. Miyata , and T. Iwanaka , “National Clinical Database (NCD) in Japan for Gastroenterological Surgery: Brief Introduction,” Ann Gastroenterol Surg 1, no. 2 (2017): 80–81. 29863115 10.1002/ags3.12026 PMC5881337

[13] S. Zhao , Y. Zhao , S. Liu , C. Zhang , and X. Wang , “Conditional Survival After Surgical Resection of Primary Retroperitoneal Tumors: A Population‐Based Study,” Cancer Cell International 21, no. 1 (2021): 60. 33472625 10.1186/s12935-021-01751-z PMC7816497

[14] K. Giuliano , N. Nagarajan , J. K. Canner , et al., “Predictors of Improved Survival for Patients With Retroperitoneal Sarcoma,” Surgery 160, no. 6 (2016): 1628–1635. 27495850 10.1016/j.surg.2016.05.041

[15] F. Tirotta , M. G. Fadel , J. Hodson , et al., “Association Between Ageing and Short‐Term Survival Outcomes in Patients Undergoing Surgery for Primary Retroperitoneal Sarcoma,” Annals of Surgical Oncology 29, no. 12 (2022): 7320–7330. 35854029 10.1245/s10434-022-12231-7

[16] K. H. Wilkinson , C. G. Ethun , M. Hembrook , et al., “Outcomes of Elderly Patients Undergoing Curative Resection for Retroperitoneal Sarcomas: Analysis From the US Sarcoma Collaborative,” Journal of Surgical Research 233 (2019): 154–162. 30502242 10.1016/j.jss.2018.07.050

[17] Q. Guo , J. Zhao , X. Du , and B. Huang , “Survival Outcomes of Surgery for Retroperitoneal Sarcomas: A Systematic Review and Meta‐Analysis,” PLoS One 17, no. 7 (2022): e0272044. 35901187 10.1371/journal.pone.0272044 PMC9333279

[18] C. Abatini , L. Barberis , C. Lodoli , et al., “Impact of Severe Postoperative Complications and P‐POSSUM Score on Oncological Outcomes in Primary Retroperitoneal Sarcoma: Insights From a Tertiary Cancer Center,” Cancers (Basel) 17, no. 11 (2025): 1787. 40507268 10.3390/cancers17111787 PMC12153596

[19] L. M. Berclaz , S. I. Goldberg , S. Cohen , et al., “Preoperative Radiation Therapy Is Not Associated With Postoperative Complications in Patients With Retroperitoneal Sarcoma,” Annals of Surgical Oncology 32, no. 3 (2025): 1522–1528. 39570298 10.1245/s10434-024-16584-z

[20] A. J. MacNeill , A. Gronchi , R. Miceli , et al., “Postoperative Morbidity After Radical Resection of Primary Retroperitoneal Sarcoma: A Report From the Transatlantic RPS Working Group,” Annals of Surgery 267, no. 5 (2018): 959–964. 28394870 10.1097/SLA.0000000000002250

[21] M. J. Maurice , J. M. Yih , J. B. Ammori , and R. Abouassaly , “Predictors of Surgical Quality for Retroperitoneal Sarcoma: Volume Matters,” Journal of Surgical Oncology 116, no. 6 (2017): 766–774. 28608360 10.1002/jso.24710

[22] S. J. Judge , K. Lata‐Arias , M. Yanagisawa , et al., “Morbidity, Mortality and Temporal Trends in the Surgical Management of Retroperitoneal Sarcoma: An ACS‐NSQIP Follow Up Analysis,” Journal of Surgical Oncology 120, no. 4 (2019): 753–760. 31355444 10.1002/jso.25649

[23] E. Z. Keung , Y. J. Chiang , J. N. Cormier , et al., “Treatment at Low‐Volume Hospitals Is Associated With Reduced Short‐Term and Long‐Term Outcomes for Patients With Retroperitoneal Sarcoma,” Cancer 124, no. 23 (2018): 4495–4503. 30317543 10.1002/cncr.31699 PMC6289614

[24] R. Schmitz , M. A. Adam , and D. G. Blazer, 3rd , “Overcoming a Travel Burden to High‐Volume Centers for Treatment of Retroperitoneal Sarcomas Is Associated With Improved Survival,” World Journal of Surgical Oncology 17, no. 1 (2019): 180. 31684956 10.1186/s12957-019-1728-z PMC6829854

[25] D. Callegaro , C. P. Raut , D. Ng , et al., “Has the Outcome for Patients Who Undergo Resection of Primary Retroperitoneal Sarcoma Changed Over Time? A Study of Time Trends During the Past 15 Years,” Annals of Surgical Oncology 28, no. 3 (2021): 1700–1709. 33073340 10.1245/s10434-020-09065-6

[26] D. P. Nussbaum , C. N. Rushing , W. O. Lane , et al., “Preoperative or Postoperative Radiotherapy Versus Surgery Alone for Retroperitoneal Sarcoma: A Case‐Control, Propensity Score‐Matched Analysis of a Nationwide Clinical Oncology Database,” Lancet Oncology 17, no. 7 (2016): 966–975. 27210906 10.1016/S1470-2045(16)30050-X

[27] F. Tirotta , A. Bacon , S. Collins , et al., “Primary Retroperitoneal Sarcoma: A Comparison of Survival Outcomes in Specialist and Non‐Specialist Sarcoma Centres,” European Journal of Cancer 188 (2023): 20–28. 37178646 10.1016/j.ejca.2023.04.004

[28] M. A. Adam , D. Moris , S. Behrens , et al., “Hospital Volume Threshold for the Treatment of Retroperitoneal Sarcoma,” Anticancer Research 39, no. 4 (2019): 2007–2014. 30952744 10.21873/anticanres.13311

