# Supplementary material for: Real‐World Data of Retroperitoneal Tumor Surgeries Performed by Gastroenterological Surgeons in Japan: Analysis Based on the National Clinical Database
Source: Ann Gastroenterol Surg. 2026 Feb 24;10(4):1331–6. doi: 10.1002/ags3.70204 (PMC13326821; doi:10.1002/ags3.70204)
Supplement: Supplementary file 1 — Figure S1: Number of surgeries by age group per year. Temporal trends in the number of surgeries stratified by age group over the study period (2019–2021). Figure S2: Postoperative complication rates by surgical procedure. Comparison of postoperative complication rates across six major surgical procedures. [file AGS3-10-1331-s001.docx]

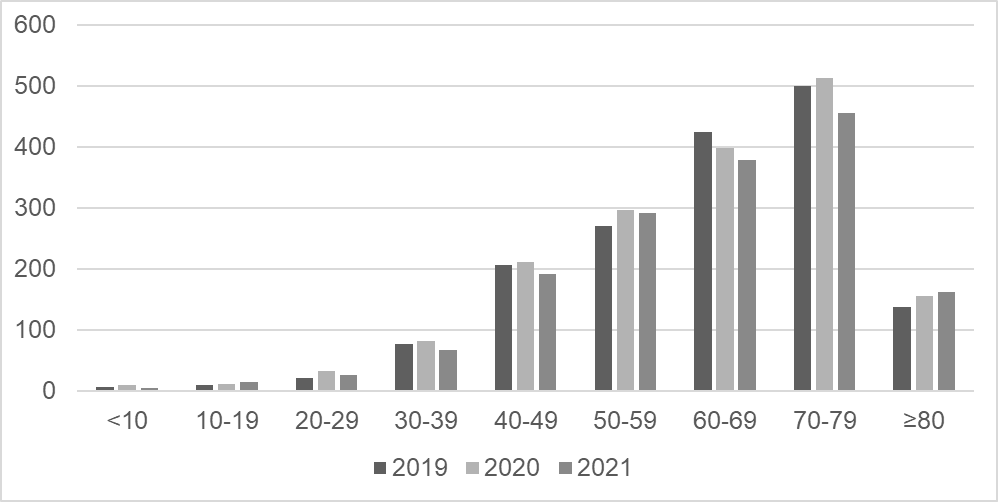


Supplemental fig.1 : Number of surgeries by age group per year


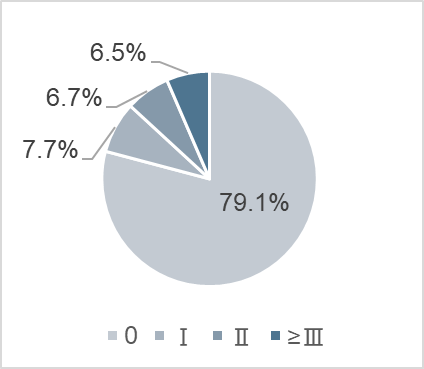


Supplemental fig.2 : Postoperative complications by surgical procedure


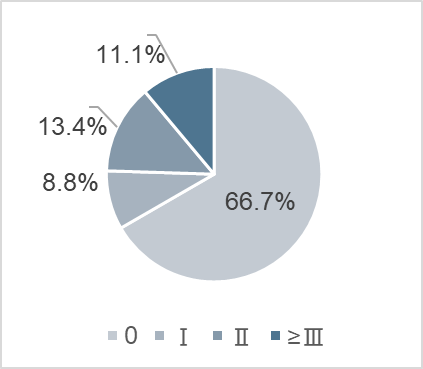

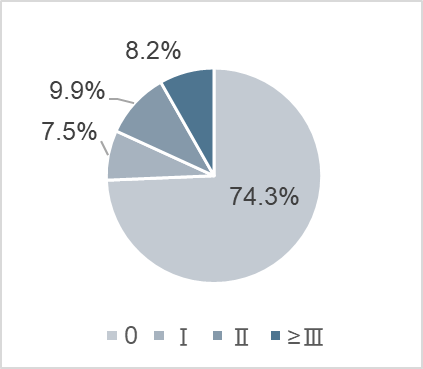


(a)OQ0094

(b) OQ0095

(c) OZ0008


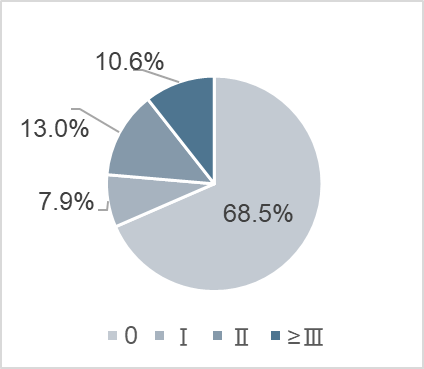

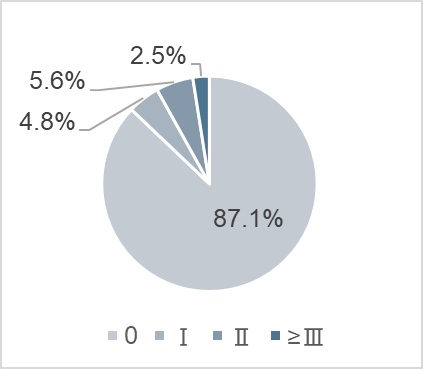

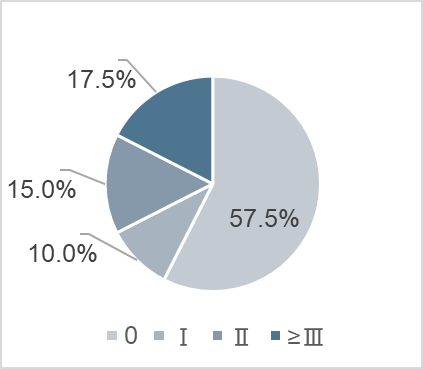


(d) OZ0009

(e) SZ0010

(f) NQ0755

Supplemental table 1: Number of surgeries by NCD procedure code (2019 and 2021)

| Code | Operative procedure | Total | benign | malignant |
| --- | --- | --- | --- | --- |
| OQ0094 | Omental, mesenteric, and retroperitoneal tumor removal without intestinal resection | 1703 | 657 | 1046 |
| OQ0095 | Omental, mesenteric, and retroperitoneal tumor removal with intestinal resection | 355 | 52 | 303 |
| OZ0008 | Retroperitoneal malignant tumor simple resection | 1458 | 160 | 1298 |
| OZ0009 | Retroperitoneal malignant tumor radical resection | 775 | 41 | 734 |
| SZ0010 | Laparoscopic retroperitoneal tumor resection | 644 | 310 | 334 |
| NQ0755 | Multiple organ resection with peritoneal resection | 41 | 3 | 38 |
| Total |  | 4976 | 1223 | 3753 |

Supplemental table 2: Intraoperative characteristics by NCD procedure code

| Code | OQ0094  (n = 1703) | OQ0095  (n = 355) | OZ0008  (n = 1458) | OZ0009  (n = 775) | SZ0010  (n = 644) | NQ0755  (n = 41) |
| --- | --- | --- | --- | --- | --- | --- |
| Operation time,  min [IQR] | 171  [112, 264] | 272  [177, 373] | 194  [128, 282] | 283  [192, 404] | 151  [101, 235] | 337  [221, 489] |
| Blood loss,  ml [IQR] | 100  [10, 519] | 380  [100, 992] | 190  [50, 606] | 485  [170, 1200] | 9  [0, 50] | 710  [200, 1519] |

Supplemental table 3: Postoperative complications classified according to the Clavien-Dindo classification by age group

| Age | <10  (n = 20) | 10-19  (n = 35) | 20-29  (n = 78)) | 30-39  (n = 224) | 40-49  (n = 609) | 50-59  (n = 859) | 60-69  (n = 1201) | 70-79  (n = 1468) | ≥80  (n = 454) |
| --- | --- | --- | --- | --- | --- | --- | --- | --- | --- |
| Grade 0 | 19 (95.0) | 30 (85.7) | 70 (89.7) | 186 (83.0) | 480 (78.8) | 645 (75.1) | 897 (74.7)) | 1104 (75.2) | 334 (73.6) |
| Grade I | 1 (5.0) | 1 (2.9) | 2 (2.6) | 12 (5.4) | 43 (7.1) | 69 (8.0) | 98 (8.2) | 110 (7.5) | 30 (6.6) |
| Grade II | 0 (0) | 3 (8.6) | 5 (6.4) | 7 (3.1) | 45 (7.4) | 85 (9.9) | 118 (9.8)) | 130 (8.9) | 53 (11.7) |
| ≥ Grade III | 0 (0) | 1 (1.3) | 1 (1.3) | 19 (8.5) | 41 (6.7) | 60 (7.0) | 88 ( 8.4) | 124 (8.4) | 37 (8.1) |
